# Is Vitamin A Supplementation Campaign Still Justified? A Qualitative Study Exploring Insights From Policymakers and Programme Planners in the Democratic Republic of the Congo

**DOI:** 10.1155/jnme/3033218

**Published:** 2025-09-10

**Authors:** Landry Egbende, Mala Ali Mapatano, Silondile Luthuli, Pierre Z. Akilimali, Ingunn M. S. Engebretsen

**Affiliations:** ^1^Department of Nutrition, Kinshasa School of Public Health, University of Kinshasa, P.O. Box 11850, Kinshasa, Democratic Republic of the Congo; ^2^Centre for Rural Health, University of Kwazulu-Natal, Durban, South Africa; ^3^Patrick Kayembe Research Centre, Kinshasa School of Public Health, University of Kinshasa, P.O. Box 11850, Kinshasa, Democratic Republic of the Congo; ^4^Department of Global Public Health and Primary Care, Centre for International Health, University of Bergen, Bergen, Norway

**Keywords:** DRC, justification, policymakers, programme planners, qualitative study, relevance, vitamin A supplementation

## Abstract

**Background:** According to the last national survey in 1998, the prevalence of vitamin A deficiency (VAD) was as high as 61% in the Democratic Republic of the Congo (DRC), leading to the implementation of the vitamin A supplementation (VAS) programme. The available data are outdated and require situation analysis and vitamin A status data. While these data are missing, the relevance of the VAS programme is currently at the centre of debate, and it is important to understand how policymakers and programme planners perceive VAS. This study aims to explore the insights of policymakers and programme planners regarding its relevance and justification.

**Methods:** This study used an exploratory qualitative design. Data were collected using key informant interviews with policymakers and programme planners at the national, provincial and health zone levels, including a total of 25 participants. Data were analysed using thematic analysis.

**Results:** The participants recognised the relevance of VAS in reducing morbidity and mortality, especially in children. However, they identified challenges in communication and delivery mechanisms, such as insufficient supplement supply and a lack of provider motivation. The participants' opinions diverged regarding the justification of the VAS programme. Some participants perceived VAS as an essential strategy due to its benefits, while others advocated for alternative, cost-effective approaches, such as improving vitamin A dietary intake through fortification.

**Conclusion:** Although VAS was considered important, our findings highlight the need to investigate alternative approaches, such as promoting affordable vitamin A–rich foods. There is an urgent need for more objective information regarding vitamin A status in the child population, and further research on VAD is necessary to evaluate the impact of the VAS programme.

## 1. Background

Vitamin A deficiency (VAD) is a major public health problem, particularly in low-income countries, where it is responsible for high morbidity and mortality in children under 5 years old [1, 2]. Around 130 million preschool children worldwide suffer from VAD, particularly in sub-Saharan Africa and Southeast Asia. In the Democratic Republic of the Congo (DRC), VAD may also be a public health problem [3–5]. According to the last national survey in 1998, the prevalence of VAD was as high as 61%, while it was 58% in the Kasaï-Oriental province [6].

Given its important role in preventing severe morbidity and mortality from infectious diseases in children, such as measles and acute respiratory infections [7], WHO has been promoting several strategies to ensure the availability, access and utilisation of vitamin A, including promoting vitamin A–rich food intake, food fortification and vitamin A supplementation (VAS) through routine supplementation and campaigns, especially in settings with a high prevalence of VAD [8–11].

In the DRC, the VAS programme has been organised throughout the country as a twice-yearly campaign in the community and during immunisation sessions in health facilities [6]. However, routine VAS is not yet effective nationwide. The fortification of certain foods, such as beans and corn, is organised piecemeal in certain regions to combat VAD, so far dependent on the support of certain partners [12].

VAS is a strategy that is currently at the centre of debate, both in its use and organisation. In some countries, the implementation of the VAS programme has been assessed, and cases of hypervitaminosis A have been found in some areas due to supplementation [13]. In other countries, such as South Sudan, after an evaluation of the VAS programme, the strategy was still justified but with some suggestions for improving delivery and communication strategies [14]. In contrast, in other countries, such as South Africa, evaluations of VAS have identified cases of hypervitaminosis A in certain regions, prompting the discontinuation of the VAS programme [15, 16]. Therefore, the promotion of vitamin A consumption through diet is increasingly seen in many countries as an effective strategy to combat VAD [17–20].

In the DRC, at the national level, VAS is the only strategy organised throughout the country, although its actual effect on reducing VAD remains unclear. Monitoring of vitamin A levels and programme delivery has been scarce since its implementation more than two decades ago. Objective and robust data for decision makers are urgently needed. In the absence of such data for DRC, policymakers still have to make decisions based on uncertainties involving data from other contexts, old data or case information. In light of this uncertainty, this study aims to explore the thoughts and insights of policymakers and programme planners involved in VAS in the DRC regarding its implementation, perceived relevance and justification.

## 2. Methods

### 2.1. Study Design

An exploratory qualitative design using key informant interviews (KIIs) was employed to explore insights from policymakers and programme planners at various levels of the DRC health system on the relevance and justification of the VAS approach. The study presentation adheres to the Consolidated Criteria for Reporting Qualitative Research (COREQ) guideline [21]. The study was part of a project assessing VAS from the leadership, implementer and receiver perspectives while also gathering objective vitamin A serum-level evidence in a selected smaller population.

### 2.2. Study Setting

The health system is structured into three levels: the central (national) level, the provincial level and the operational (health zone) level. The central level is the normative level, where all decisions and strategies are formulated. The provincial level is responsible for technical supervision and monitoring and translating directives, strategies and policies into instructions to facilitate their implementation at the health zone level. The health zone level serves as the operational level, responsible for implementing the primary healthcare strategy [22].

This study was conducted with policymakers and programme planners at the national, provincial and health zone levels. Kasai-Oriental was selected at the provincial level as it is one of the regions most severely affected by hunger and food insecurity. Forecasts from the integrated food and nutrition security analysis conducted in 2023 indicate that the percentage of the population in Kasai-Oriental experiencing food insecurity will rise in the coming years, primarily due to the Kamwina Nsapu conflict [23]. The province of Kasai-Oriental has 19 health zones, 10 of which are urban and 9 rural. We aimed to work in approximately a quarter of these health zones and approached five health zones. As urban areas constitute 53% of health zones, three urban health zones and two rural ones were selected [23].

### 2.3. Sampling and Recruitment

All participants in this study were selected purposively. At the national and provincial levels, participants were identified by targeting key stakeholders in the nutrition sector. Based on their availability and willingness, they were invited to participate in the study.

At the national level, we included participants from the public sector, including the Ministry of Health and the Ministry of Agriculture, as well as international organisations working in nutrition and academics specialising in nutrition. Participants were required to have a minimum of 5 years of experience working in nutrition or VAS to be eligible for inclusion in our study.

Within Kasai-Oriental, we included the health zone management team responsible for implementing VAS. This team comprised the health zone officer, the nutritionist and the community health facilitator.

In total, 25 stakeholders were included in our study: 8 at the national level, 2 at the provincial level and 15 at the health zone level (Table 1).

### 2.4. Data Collection

All KIIs were conducted face-to-face using a pretested semistructured interview guide in French. The guide covered topics including VAS and its need, vitamin A delivery approach, communication strategy, challenges in implementing VAS and strategies used in the community to facilitate its uptake.

To ensure privacy, interviews were conducted at participants' respective offices using two audio recorders. The interviews started with a succinct introduction outlining the study objective, followed by a presentation of the participant, emphasising their nutritional experience. Authorisation to record the conversation was sought after obtaining the participant's consent to participate in the study.

After the interview, the recordings were deidentified, password protected and archived in a folder on the computer, with a backup copy stored on a drive to prevent potential loss. In addition to recording the interviews, detailed field notes were taken to capture additional observations.

Interviews with participants averaged 45 min and were conducted from July 15 to August 15, 2024, totalling 25 face-to-face interviews. All interviews were carried out by the first author, a medical doctor and public health specialist focussing on nutrition, with extensive experience in qualitative data collection.

### 2.5. Data Analysis

The transcripts underwent an inductive thematic analysis following the methodology of Braun and Clarke, as it aligns with exploratory studies where existing theories may be insufficient to fully capture the complexity of the data [24]. All KIIs were transcribed verbatim, and the transcripts were reviewed and supplemented with field notes. The first author translated the transcripts from French to English.

During the initial phase of data familiarisation, the first author listened to the audio recordings and meticulously reviewed the interview transcripts multiple times to deeply engage with the content. After this phase, the coding process began using NVivo 14 for all transcripts. This process was completed by two researchers, who independently coded the transcripts. A discussion was then held with the whole team on the proposed codes, and an agreement was reached on which codes should be used.

The final list of codes was established after refining the initial ones. Related codes exhibiting similar patterns or characteristics were grouped together to establish preliminary themes. These emerging themes were then presented and discussed for adoption with all co-authors.

The preliminary themes underwent multiple rounds of deliberation, restructuring and refinement to enhance depth and coherence, ensuring that each topic maintained a distinct focus centred around a single primary idea.

### 2.6. Reflexivity

The first author of this study is a medical doctor and a public health specialist with a focus on nutrition. He is a PhD candidate and has long been involved in qualitative research in various provinces of the country. In the context of this research, the first author was responsible for conceptualisation and participated in data collection by conducting interviews with various key informants. The participants were free to express their points of view, and data collection was organised to ensure neutrality in responses. To validate the data, the first author summarised the participants' main answers during the interview before moving on to the next question, allowing participants to confirm that their responses had been accurately captured. The data analysis was conducted by a multidisciplinary team from various research institutions, including medical doctors with a focus on nutrition, as well as experts in social sciences. The first author's full involvement in data collection allowed him to become more familiar with the data, while regular research team meetings ensured a shared understanding among all members.

### 2.7. Ethical Considerations

Ethical approval was obtained from the Ethics Committee of the School of Public Health at the University of Kinshasa (reference number: ESP/CE/67/2024) and the Norwegian Institutional Review Board (REK West) (reference number: 645696).

The four ethical principles outlined in the Declaration of Helsinki—autonomy, confidentiality, beneficence and nonmaleficence—were acknowledged. Autonomy was preserved, as participants granted verbal informed consent following a comprehensive explanation of the study's objectives. Participants were informed that their participation was voluntary and that they could withdraw from the conversations at any time. Confidentiality was ensured by removing all identifying information from transcripts and assuring participants that their responses would remain confidential.

## 3. Results

The results of this study are organised into four themes: (1) contextual understanding of the VAS programme in the DRC, (2) challenges in implementing the VAS strategy, (3) strategies within the community to promote VAS and (4) whether the implementation of VAS is justifiable.

### 3.1. Contextual Understanding of the VAS Programme in the DRC

This theme, describing the organisation of the VAS in the DRC, was subdivided into two subthemes: description of the VAS approach and VAS as part of routine activities.

#### 3.1.1. Description of the VAS Approach

Most participants widely described VAS in terms of its importance. It was presented as an important WHO strategy for combating VAD in countries with a high prevalence of the condition.

VAS is a strategy of the World Health Organization to combat Vitamin A deficiency among children under 5 years old. It is an effective strategy for proper child growth. The target population is children under 5 years old; 100,000 units are administered to children aged 6–11 months and 200,000 units to those over 11 months, with the supplementation occurring every 6 months. (KII, 03, National level)

In addition, various supplementation approaches were used in the health zones. According to participants, VAS is organised as a campaign twice a year, generally in the middle and at the end of the year, but it is also routinely conducted in health facilities. Participants also noted that, for some years, VAS has been coupled with a deworming campaign using albendazole to combat intestinal worms in children. For cost reasons, VAS is sometimes integrated into the polio vaccination campaign for children.

In our health area, we organise supplementation in several ways. We conduct mass campaigns, especially in March. Additionally, there is a routine approach where every child receives vitamin A at 6 months and again at 12 months at health centres. (KII, 13, Health zone level)

However, it is sometimes combined with polio vaccination campaigns, and we've found that combining vitamin A supplementation with polio vaccination increases parental acceptance of the polio vaccine and reduces refusals. (KII, 03, Health zone level)

#### 3.1.2. VAS as Part of the Routine Activities

Child immunisation visits take place routinely in health facilities according to the DRC immunisation schedule. Children receive a certain number of vaccines up to the age of two and must, however, visit health facilities every month for this purpose. Some participants also described these routine immunisation visits as a promoted approach for VAS.

In the DRC, VAS is carried out twice a year for children aged 6–19 months. It is done either through campaigns or routinely during preschool consultations. (KII, 08, National level)

For the participants, the door-to-door campaign strategy had some challenges, such as the absence of children during the campaign period, and the routine approach was seen as a solution. They described the routine approach as one that would take place both in the community with community health workers (CHWs) and in health facilities with providers during routine immunisation visits. However, this approach was described as not yet truly effective due to insufficient doses of vitamin A, making it almost similar to the campaign strategy.

We aim to make supplementation more routine, but it is not yet working as intended. We are also experimenting with community-based routine supplementation through community health workers with Helen Keller, but this still resembles mass campaigns. We have not yet achieved a true routine system. (KII, 01, National level)

Intensive community-based routinisation is a strategy adopted by the country to provide vitamin A. Previously, it was administered during mass campaigns. Now, we aim to integrate it into routine activities. Although routinisation is the term used, it means children receive vitamin A every six months. It's a concept bridging campaigns, aiming to make vitamin A continuously available. When a child experiences diarrhoea, respiratory infections, or prolonged fevers, they should receive vitamin A as part of routine care. We're working towards making this effective and consistent. (KII, 01, Provincial level)

### 3.2. Challenges in Implementing the VAS Strategies

The participants emphasised that supplementation has its place in the country, given its many benefits in reducing morbidity and mortality, especially among children under five. However, it faces several challenges in ensuring full coverage of children across the country and preventing VAD. These challenges include the lack of knowledge of VAS among CHWs, the low supply of supplements and funding constraints affecting VAS implementation.

#### 3.2.1. Lack of Knowledge on VAS of CHWs

According to the participants, CHWs are responsible for administering vitamin A during the various campaigns that are organised. In addition, CHWs must convey the right message to the population in clear, simple terms to facilitate adherence. However, participants perceived that this role is not always well executed, as CHWs sometimes convey messages that are not well adapted, making it more difficult for mothers to accept VAS.

While the community health workers have some knowledge about vitamin A, their communication can be weak. Often, households do not receive sufficient information on the full benefits of vitamin A beyond its role in vision. Additional training for CHW is necessary to improve their effectiveness in disseminating information. (KII,01, Provincial level)

Yes, strengthening the capacity of community health workers is crucial. Sometimes, community health workers convey incorrect messages or make unfulfilled promises, which can damage the credibility of supplementation. (KII, 03, Health zone level)

#### 3.2.2. Low Supplies

The participants recognised that the availability of vitamin A supplements is crucial for the success of VAS. For them, without these inputs, the supplementation campaign cannot take place, or if it does, many children may miss out on receiving the supplement. Nonetheless, the participants indicated that the availability of these supplies remains problematic in various health zones, particularly regarding the quantity or doses of vitamin A, hindering the effective execution of the programmes.

In my opinion, I suggest ensuring that supplements are available on time. Even outside of campaigns, having them available in the health zone can help with the growth of targeted children. (KII, 09, Health zone level).

The biggest challenge is the availability of supplements because, despite multiple approaches, without enough supplements, the goal will never be achieved. (KII, 03, National level)

#### 3.2.3. Funding as a Challenge to VAS Implementation

VAS activities require a large number of staff, but the most important are the CHWs and providers who administer the supplements to the children, according to the participants. In addition, the work is very demanding, as all the children in the area need to be supplemented. The participants stated that CHWs are not well remunerated despite their heavy workload. Most participants perceived this as a significant obstacle to the successful execution of VAS, particularly when this strategy is occasionally integrated with other campaigns, such as the polio vaccine campaign.

It's crucial to motivate the staff involved in supplementation campaigns. The work is demanding, and staff need proper incentives. The state should ensure that workers are well-compensated and supported. (KII,11, Health zone level)

The participants emphasised that door-to-door campaign activities generally incur high costs, as they require staff mobilisation. Staff must walk across villages or neighbourhoods to supplement the children. This strategy demands more funding than the routine supplementation approach in health facilities, which could help minimise this funding challenge.

The main challenge with the campaign approach is the cost, as this strategy requires a lot of money. The estimated goal is to reach at least 90% coverage, but there are various reasons for not achieving this. (KII, 08, National level)

Mass campaigns are more costly compared to routine supplementation, which requires strengthening the community health workers and possibly increasing public awareness. (KII,01, National level)

Additionally, according to participants, dependence on external funding is another challenge. In some sites, the withdrawal of a funding partner has negatively impacted VAS.

It should be noted that funding has suffered at times. When I started these activities, it was with Helen Keller first. We organized child follow-up days. Then there were large campaigns. After Helen Keller withdrew, UNICEF came with funding, but it was on a routine basis. The funding was reduced by around 90%. (KII, 01, Health zone level)

### 3.3. Strategies Within the Community to Promote the VAS

Despite these challenges, the participants indicated that several strategies are being implemented across various health zones to enhance community support for VAS.

#### 3.3.1. Awareness-Raising in a Health Facility

Promotional activities for vitamin supplementation take place in the various zones during the usual consultation sessions organised in health facilities. These may be preschool consultations, prenatal consultations or other hospital services. The participants stated that these are opportunities where it is easier to convey the message at a lower cost.

Moreover, during prenatal consultations, educational sessions are used to raise awareness among mothers about the benefits of vitamin A and albendazole. Themes for these sessions are chosen based on needs identified, such as children who were not supplemented in the previous month. (KII, 03, Health zone level)

#### 3.3.2. Awareness-Raising With CHWs in the Community

Participants highlighted the role of CHWs in vitamin A awareness campaigns. CHWs are responsible for raising awareness of the VAS campaign at the community level. They convey the message either by using megaphones so that the message is better heard or even late at night, allowing those who work during the day to hear it.

We use awareness campaigns. Before supplementation, we activate community dynamics. Volunteers go door-to-door and use megaphones to spread the message. They educate mothers to ensure they give vitamin A to their children. (KII, 12, Health zone level)

We also provide messages to community health workers during their home visits, showing the benefits of vitamin A to households. This has led to increased enthusiasm; now, mothers more readily accept having their children supplemented when the activity takes place. (KII, 13, Health zone level)

In addition to raising awareness during campaigns, the CHWs, who are members of the Cellule d'Animation Communautaire (CAC), a community dynamics organisation at the zone level, make visits as part of their routine activities, according to participants. They monitor the health status of the population and contribute to various health interventions, including VAS, vaccination, mosquito net distribution and passive malnutrition screening. They also share messages during routine activities, making them more familiar with the community.

The Community health workers in each health area also include the CAC. Each CAC has a president, and so on. The CAC president is supposed to visit at least three households a day. During these visits, sensitization also takes place. (KII, 15, Health zone level)

Here in Miabi, we have a community-driven approach. Community health workers are familiar with their households, and there are quite a few agents here. Each community health worker is well-acquainted with the children in their area, and during supplementation, they go around and raise awareness. (KII, 09, Health zone level)

#### 3.3.3. Using Community Leaders

Community leaders play a major role, especially in promoting health interventions and persuading the community to accept them, according to the participants. Among these leaders, both political and administrative authorities, as well as religious leaders, are involved. They are often engaged in health promotion activities, particularly where community barriers exist. Their involvement helps overcome most community challenges to VAS.

We also involve community leaders, opinion leaders, pastors, sector chiefs, and certain political and administrative authorities to help convince mothers to allow their children to receive vitamin A. (KII, 05, Health zone level)

This means involving political and administrative authorities, as well as religious leaders, to ensure they are aware of the activity. (KII, 14, Health zone level)

#### 3.3.4. Using Media

Most participants highlighted the importance of media in promoting health interventions, including VAS. In rural regions, community radio stations are the primary media outlets used to disseminate information regarding VAS.

Mass campaigns are conducted via media, including community radios, to reach a broader audience. (KII, 07, National level)

### 3.4. If the Implementation of VAS Is Justifiable or Not

Participants had different opinions on the rationale for VAS in children under 5 years old. For some, the strategy seemed appropriate, given the country's health and nutrition challenges. Conversely, another group considered the approach inappropriate and costly, advocating for the promotion of local vitamin A–rich foods.

#### 3.4.1. VAS Is Still Justified

For the participants, supplementation is important in preventing the consequences of VAD, such as infections, diarrhoeal diseases, measles and visual disorders, thereby reducing morbidity and mortality in children under five. They reported that regions where this strategy had been halted due to stock shortages saw higher rates of infectious diseases and diarrhoea in children. Restricted access to vitamin A–rich foods exacerbated these conditions.

The strategy is essential and appropriate, especially for our health zone. In the past, UNICEF provided us with a family kit that included vitamin A, but that support has stopped. We have observed that children with gastroenteritis benefit from vitamin A supplementation, which reduces the frequency of diarrheal diseases. (KII, 04, Health zone level)

Yes, the intervention is justified and necessary. As a PRONANUT representative, I can say that given the levels of malnutrition and diseases like measles, this intervention is crucial, especially since food insecurity is prevalent in the province. While we are working to educate people about obtaining vitamin A from food, the fact is that the production of such foods is often insufficient. Until people understand and access sufficient nutritional sources, the supplementation needs to continue. (KII, 01, National level)

#### 3.4.2. Promote Alternative Approaches

As vitamin A is present in local foods, promoting the consumption of vitamin A–rich local foods was perceived by some participants as an alternative approach to combating VAD. Some emphasised the importance of educating the population on how to preserve vitamin A in foods during cooking, considering this intervention easier to implement than a supplementation campaign.

We conduct many culinary demonstrations with community members, explaining that vitamin A can be found in red palm oil, but that the way they prepare their food may destroy the vitamin A content. We advise them to avoid destroying the carotene in the oil during cooking. (KII, 01, Provincial level)

Personally, I am more focused on food security. I believe we should develop our local food culture based on palm oil, improve this cuisine, and avoid spending money on vitamin A supplements. We have a more cost-effective and nutritious option available locally. (KII, 02, Provincial level)

For others, promoting agriculture to support food security would help combat VAD. Other interventions, such as fortification, would also contribute considerably.

We recommend not only providing synthetic vitamin A capsules but also promoting the intake of vitamin A through foods. Increased awareness and consumption of fortified or naturally vitamin A-rich foods are essential. I would recommend intensifying efforts to promote agriculture, especially the cultivation of vitamin A-rich foods. (KII, 01, Provincial level)

## 4. Discussion

This study explored insights from policymakers and programme planners about the VAS programme. The study highlights recognition of the importance of VAS in reducing child morbidity and mortality while also identifying significant challenges in its delivery mechanisms. However, participants had divergent opinions regarding the justification of the VAS programme. While some participants viewed VAS as an indispensable strategy given its demonstrated benefits, others advocated for alternative, cost-effective approaches, such as vitamin A intake through foods, including both vitamin A–rich foods and fortification.

VAS was perceived as an indispensable strategy due to its benefits in reducing child morbidity and mortality. Several studies have similarly noted the life-saving potential of VAS in reducing mortality among children under 5 years old [25–27]. Haselow et al. showed in their study in South Sudan that VAS was a key life-saving intervention to combat VAD, suggesting that delivery mechanisms should be improved to achieve optimal VAS coverage [28]. In Nigeria, Aghaji et al. found that there were inequalities in VAS coverage, which contributed to morbidity and mortality in children, suggesting that context-specific strategies should be put in place to increase VAS coverage and address this mortality issue [29].

We think that the lack of optimal VAS coverage and the increase in the prevalence of infectious diseases in children under the age of five explain why this strategy is seen as essential [30]. Additionally, food insecurity in most regions limits access to basic foods [31, 32].

On the other hand, VAS was seen as insufficient by some stakeholders, suggesting the promotion of other alternatives, including the consumption of vitamin A through foods, which could be contrary to WHO recommendations for VAS [33]. This is in line with several studies suggesting the implementation of sustainable interventions to combat VAD instead of short-term interventions [3, 34].

Rice et al., in their review article, described palm oil as an important source of vitamin A from the point of view of acceptability and accessibility, making it capable of reducing VAD [35]. Dong et al. found in their meta-analysis on the effect of red palm oil on VAD that palm oil was effective in preventing or alleviating VAD [36]. Vitamin A is a nutrient found in foods from both animal and plant sources, especially dark leafy vegetables and fruits [37].

These vitamin A–rich foods are available and consumed in the DRC. One of the food sources rich in vitamin A available in the country is palm oil, which is accessible to most of the population [12]. Even though excessive consumption of palm oil may be associated with cardiovascular diseases due to increased cholesterol levels, requiring caution in its intake [38–40], it remains one of the most sustainable ways of combating VAD [41–43]. However, the promotion of appropriate preparation strategies would be an intervention that facilitates the preservation of vitamin A [44].

In addition, the promotion of other foods, such as fruit and vegetables, can also be supported and used to combat deficiencies, as they have proven their effects in several countries and could help prevent dependence on international partners [45]. VAS is costly and is implemented due to support from international partners in most African countries, including the DRC, given the socio-economic context of these countries [46]. VAS also faces challenges, such as low provider motivation and geographical barriers that limit optimal coverage [47]. Vitamin A through food would be a sustainable intervention, even in the absence of support from international partners [45].

Agriculture in the DRC faces many challenges, particularly related to the lack of appropriate infrastructure, but also soil infertility and pests in certain areas [48]. Government involvement is needed to promote sustainable agriculture. In addition, promoting gardening at the household level can contribute to increasing the consumption of green leafy vegetables and combating VAD. This alternative would be less costly and would also help to support food security at the household level.

### 4.1. Policy and Practice Implications

This study reveals the perceptions of stakeholders responsible for VAS at the national level. It highlights the need to rethink VAS as recommended by WHO [33]. Although VAS is seen as an important strategy for reducing morbidity and mortality in children, the path towards more sustainable, context-sensitive interventions was also highlighted [49].

The study points towards alternative approaches based on local vitamin A–rich foods [45] and suggests gradually reducing reliance on the VAS strategy, which is more expensive and does not cover all children. In some countries, VAS has been associated with cases of hypervitaminosis in areas where there was already sufficient consumption of vitamin A–rich foods [15, 16]. In other countries, such as Nepal, VAS alone was not regarded as a crucial strategy for reducing mortality [34]. This has led to decisions such as stopping supplementation in certain regions or even suggesting a reduced dose instead of high-dose vitamin A [16, 50].

We suggest that more sustainable, low-cost strategies adapted to the context, such as promoting agriculture and good dietary practices, should be encouraged to reduce VAD in a sustainable way [20, 21, 44, 45]. VAS could be targeted at disadvantaged communities instead of being administered uniformly to all children [51]. Assessing the prevalence of implementation of and compliance to VAS [52], vitamin A serum levels and VAD is urgently needed to guide policy and decision-making as the last national VAD survey was conducted nearly 30 years ago. Revising the VAS policy based solely on the perceptions of policymakers and programme planners is inadequate and could fail to address the root causes of VAD. Research is also needed evaluating complementary nutrition interventions—such as exclusive breastfeeding practices and dietary intake of vitamin A–rich foods—that contribute to improving vitamin A status in children. Studies on implementation aspects such as delivery, reach, costs and sustainability is also warranted. While our project is including a vitamin A assessment in a selected smaller population and will be informative, that cannot replace the much larger need for multidisciplinary country representative data.

### 4.2. Limitations and Strengths

Our study has some limitations. It should be noted that the level of information at the provincial level was limited to the province of Kasai-Oriental, and we chose to work in only five health zones. Therefore, the information gathered cannot be generalised to the entire province or country. As this is a qualitative study, it cannot, on its own, lead to a policy change without a prior assessment of VAD prevalence. That said, this is not the role of qualitative exploratory methods but rather to provide insights into perceptions and experiences. In addition, we took care to include the main stakeholders and partners involved in implementing VAS in the country, as they are at the centre of this activity.

## 5. Conclusion

VAS is a crucial approach to addressing VAD in the DRC, considering the social background of illness, mortality and malnutrition, particularly among children under 5. Furthermore, addressing the challenges in VAS implementation and exploring alternative approaches will be essential for achieving national and global targets for child health and nutrition, including the Sustainable Development Goals. Further studies should focus on assessing VAD and understanding community perspectives on the VAS strategy, which has been in use for several years. Country representative data on vitamin A status and VAS implementation are warranted and should be the basis for stakeholders' decisions. In addition, additional research on the consumption patterns of vitamin A–rich foods will be important for informing national nutrition strategies.

## Figures and Tables

**Table 1:** Characteristics of the study participants.

	All (*n* = 25)	National (*n* = 8)	Provincial (*n* = 2)	Health zone (*n* = 15)
Age, median (min-max)	48 (29–70)	54 (29–70)	61 (54–68)	44 (35–68)
Sex				
Male	19 (66.0%)	7 (87.5%)	2 (100.0%)	20 (80.0%)
Female	6 (24.0%)	1 (12.5%)		5 (20.0%)
Years of experience				
< 10 years	13 (52.0%)	3 (37.5%)		10 (40.0%)
> 10 years	12 (48.0%)	5 (62.5%)	2 (100.0%)	15 (60.0%)
Type of organisation				
Public sector	21 (84.0%)	4 (50.0%)	2 (100.0%)	25 (100.0%)
Academia	2 (8.0%)	2 (25.0%)		
International organisation	2 (8.0%)	2 (25.0%)		

## Data Availability

The data that support the findings of this study are available upon request from the corresponding author. The data are not publicly available due to privacy or ethical restrictions.

## Nomenclature

CAC: Cellule d'Animation Communautaire
CHW: Community Health Worker
DRC: Democratic Republic of Congo
KII: Key Informant Interview
VAD: Vitamin A deficiency
VAS: Vitamin A supplementation
WHO: World Health Organization

## Supporting Information

Supporting Information 1 Supporting Information 1. The code tree describing the various codes, subthemes and themes.

Supporting Information 2 Supporting Information 2. The consent form used prior to the interview.

Supporting Information 3 Supporting Information 3. The key informant interview guide.
