# Supplementary figures and images for: Is Vitamin A Supplementation Campaign Still Justified? A Qualitative Study Exploring Insights From Policymakers and Programme Planners in the Democratic Republic of the Congo
Source: J Nutr Metab. 2025 Sep 10;2025:3033218. doi: 10.1155/jnme/3033218 (PMC12443511; doi:10.1155/jnme/3033218)

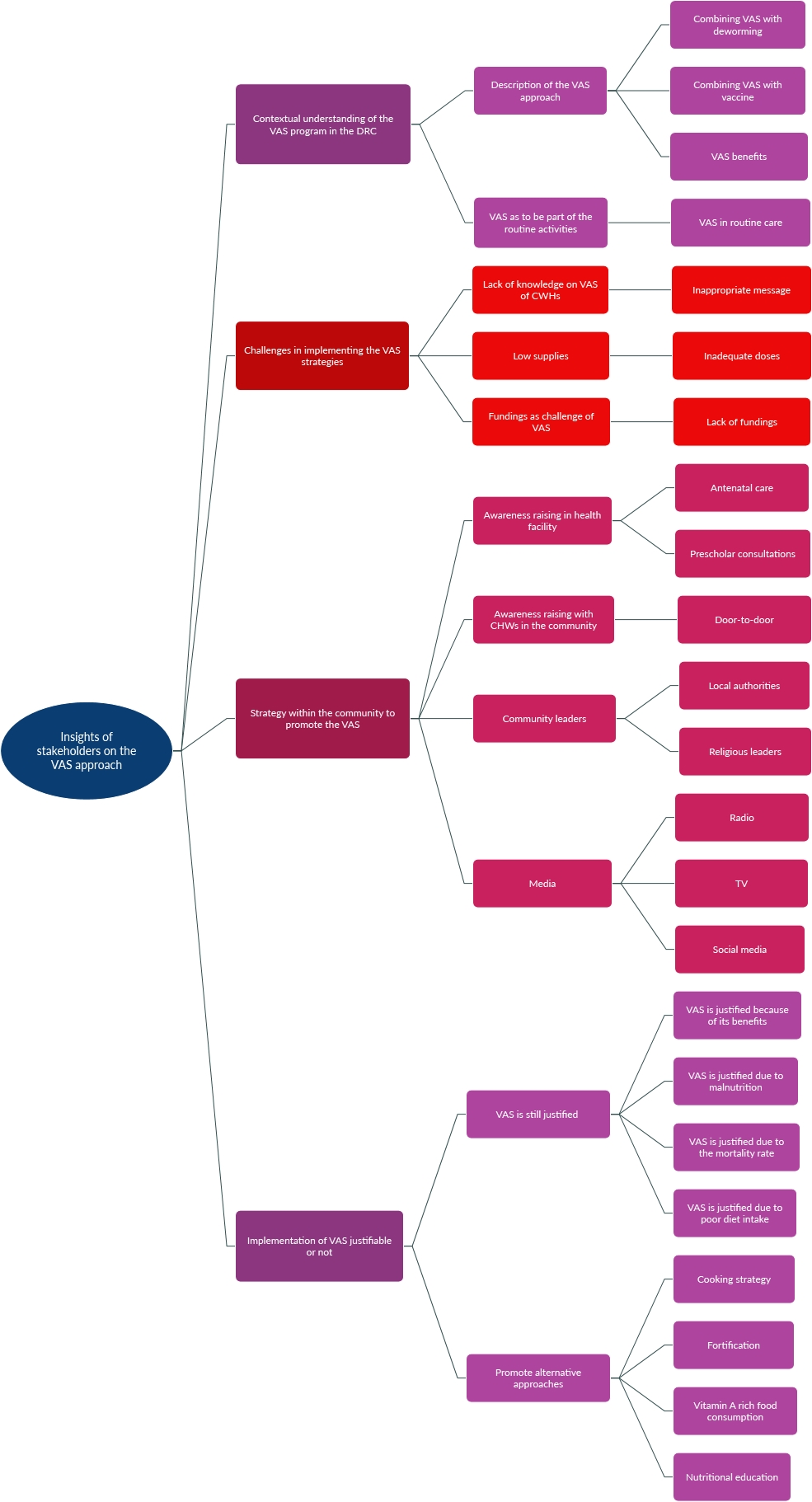

Supplement: Supporting Information 1 — The code tree describing the various codes, subthemes and themes. [file 3033218.f1.jpg]
