# Supplementary material for: Is Vitamin A Supplementation Campaign Still Justified? A Qualitative Study Exploring Insights From Policymakers and Programme Planners in the Democratic Republic of the Congo
Source: J Nutr Metab. 2025 Sep 10;2025:3033218. doi: 10.1155/jnme/3033218 (PMC12443511; doi:10.1155/jnme/3033218)
Supplement: Supporting Information 2 — The consent form used prior to the interview. [file 3033218.f2.docx]

**PARTICIPANT'S INFORMATION AND CONSENT FORM SUB STUDY 1**

**Project overview**

Vitamin A deficiency is a major public health problem in developing countries. Several strategies have been implemented to combat vitamin A deficiency, including vitamin A supplementation through organized campaigns. However, in certain contexts, this strategy has been responsible for hypervitaminosis A, whereas in certain regions, after evaluation, it should remain the case, but with an improved delivery and communication strategy. Supplementation is also implemented in our country. Two strategies exist, one in health facilities and the other in the community. The effectiveness of these two strategies in reducing deficiency should be evaluated. Furthermore, it would be important to evaluate these strategies in order to understand what is not working, to propose solutions and to guide policies. Surveys of the prevalence of vitamin A deficiency are costly, and it is often difficult to get an idea of the scale of the problem despite the strategies in place, hence the importance of this study.

**Aim of the study**

The aim of this study is to evaluate the implementation of vitamin A supplementation and the effectiveness of this intervention in food-insecure provinces in order to propose solutions for improving this strategy and guiding policies for the well-being of the population.

**About the participants**

This study will involve policy and program planners working in Health and nutrition with a focus on Vitamin A supplementation at all the level of the Ministry of Health to understand their perceptions on Vitamin A supplementation strategies in DRC. This information will be obtained by IDIs that will be conducted in Kinshasa (National level) and in two provinces (Kasai oriental and Kwango) with policy and program planners including Ministry of Health Programs, UN agencies, International NGOs, Provincial Health Division of Health and Civil Society Organization.

**Your rights as a participant**

Your participation in this study is voluntary. You may refuse to answer any question by asking to skip the question, and may withdraw at any time without further explanation.

**Risks and benefits**

There are no known or anticipated risks associated with participation in this study. The information gathered in this study will contribute to improving the health of children under 5 years of age, who are most concerned by vitamin A supplementation. We recognize that your time is valuable, but your answers will be useful in helping to improve strategies to combat vitamin A deficiency in this country.

**Confidentiality and privacy**

The information you share will be kept confidential, and only the research team involved will have access to the data. The research team will store the recorded audio and transcribed data securely in password-protected files. All information that could identify you will be removed and replaced by a coded number.

**Use of collected data**

The data will be used to obtain a PhD in public health, and scientific articles will be published in scientific journals. In addition, at the end of the research, a presentation will be made in your respective provinces to inform you of the results. Political decision-makers will also be informed of the results of our research to help improve the strategies used for the well-being of the population.

**Compensation**

There will be no financial compensation for participating in the study.

**Contact**

If you have any questions about this research study, please send an e-mail or call

Dr Egbende Mongondi Landry

[egbendelandry@gamil.com](mailto:egbendelandry@gamil.com)

+243812960837

Kinshasa School of Public Health

**B. CONSENTEMENT**

I read (or they read to me) the information about the purpose of the study, which was to evaluate the effectiveness of vitamin A supplementation in reducing the prevalence of vitamin A deficiency in children under 5 years of age in the DRC, in a language I could understand. I had the opportunity to put all my questions to the members of the research team. The answers were provided in a language I could understand. I have understood the advantages and disadvantages of participating in this study. I agree to participate in this research project under the conditions stated herein. A signed and dated copy of this information and consent form will be given to me.

**_________________________ _____________________ _________________**

**Name of participant (Initials) Signature Date**
