# Supplementary material for: Is Vitamin A Supplementation Campaign Still Justified? A Qualitative Study Exploring Insights From Policymakers and Programme Planners in the Democratic Republic of the Congo
Source: J Nutr Metab. 2025 Sep 10;2025:3033218. doi: 10.1155/jnme/3033218 (PMC12443511; doi:10.1155/jnme/3033218)
Supplement: Supporting Information 3 — The key informant interview guide. [file 3033218.f3.docx]

**Key Informant Interview Guide for Policy/Program Actors (Sub study 1)**

**Introduction and warm up**

I am a Researcher at Kinshasa School of Public Health, My name is .................., I will like to know what program planners or project managers think about vitamin A supplementation program in DRC. In order to do this, I have identified some participant groups that include policy and program planners. You are one of the people invited to participate in this study. I will like to conduct an interview with you. An interview is when I talk to people one-on-one on a topic like what I am about to start now. What I will like to know is what you think about the topic of vitamin A supplementation or how you feel about it. There are no wrong or right answers, it is just how you see the topic or the way you feel about it. Do not think that any information is too small or not relevant. I will use whatever I find out from people I speak with to advise the government on how they plan vitamin A supplementation program.

**Seek participants consent to record the session**

I will like to take note as I speak to you so that I can remember everything we discuss after I leave this place. Also because I may not be able to write very fast everything you are saying I will like to request your permission to record everything we discuss. This will not be used against you at any time and what you said cannot be traced to you. It is just to ensure that I do not miss out important information. Can I record our discussion using this device (show a digital voice recorder to the respondent)?

**Part 1. Vitamin A Supplementation implementation**

1. What do you know about vitamin A supplementation (VAS)? (Probe for the age of children who should receive VAS, the dosage of VAS for the target age group, Who provides vitamin A to children , What do you think are the benefits of VAS? What do you think are the problem that may arise if a child lacks vitamin A?.

2. What are the ways through which VAS is currently promoted in DRC? (probe for a routine delivery, use of private health facilities, campaign strategies) ? What is the best approach for you and why ? what approach cost a lot and why ? What do you think are the main challenges of the VAS delivery approaches? Do you think that the VAS is still justified in DRC ? (Why, why not ?)

3. How do you or your team promote the VAS program? (Probe for materials used to promote VAS, available communication materials such as jingle scripts, posters, banners).

**Part 2. Barriers and facilitators to VAS utilization**

3. Can you describe some of the factors that you think make some children miss VAS? (Probe for cultural factors, religious reasons, some myth or other barriers)

4. What are the things you think make mothers to receive vitamin A for their children? (Probe How can you describe the effectiveness of the VAS communication approach? What do you think can be done to improve communication about VAS?)

**Part 3. Proposed solutions to promote VAS**

5. How best do you think the VAS program can be promoted at all levels? (Probe for what government, health workers or other stakeholders can do).

**Other issues relevant to VAS**

6. Are there other things you think are important about VAS that you will like to share?

Wrap Up, Thank Respondents & Close.
